# Associations between ultrasound screening findings and cholangiocarcinoma diagnosis in an at-risk population

**DOI:** 10.1038/s41598-022-17794-9

**Published:** 2022-08-06

**Authors:** Kavin Thinkhamrop, Narong Khuntikeo, Nittaya Chamadol, Apiporn T. Suwannatrai, Surachai Phimha, Matthew Kelly

**Affiliations:** 1 Cholangiocarcinoma Screening and Care Program (CASCAP), Faculty of Medicine, Khon Kaen University, Khon Kaen, Thailand; 2 Cholangiocarcinoma Research Institute (CARI), Khon Kaen University, Khon Kaen, Thailand; 3 Data Management and Statistical Analysis Center (DAMASAC), Faculty of Public Health, Khon Kaen University, Khon Kaen, Thailand; 4 Health and Epidemiology Geoinformatics Research (HEGER), Faculty of Public Health, Khon Kaen University, Khon Kaen, Thailand; 5 Department of Surgery, Faculty of Medicine, Khon Kaen University, Khon Kaen, Thailand; 6 Department of Radiology, Faculty of Medicine, Khon Kaen University, Khon Kaen, Thailand; 7 Department of Parasitology, Faculty of Medicine, Khon Kaen University, Khon Kaen, Thailand; 8 Department of Public Health Administration, Health Promotion, and Nutrition, Faculty of Public Health, Khon Kaen University, Khon Kaen, Thailand; 9 Department of Global Health, National Centre for Epidemiology and Population Health, Australian National University, Canberra, Australia

**Keywords:** Cancer epidemiology, Cancer imaging, Cancer screening, Cancer, Risk factors, Epidemiology

## Abstract

The rate of cholangiocarcinoma (CCA) is increasing every year, especially in northeastern Thailand. Screening for CCA using ultrasonography (US) is the fastest technique to identify patients in early stage of CCA development. Currently, few studies have examined patterns of hepatobiliary abnormalities identified using US, which can be indicative of CCA. We aim to evaluate the rate of CCA and its relations to history of US findings. Our study included participants who underwent US and pathological diagnosis of CCA from the Cholangiocarcinoma Screening and Care Program, Northeast Thailand between 2013 and 2020. Data on histological findings were based on the standard protocol of the tertiary hospital at Khon Kaen University. CCA data is categorized into two groups (yes/no) to find the relationship with history of US findings. The adjusted odds ratio (AOR) and their 95% confidence intervals (CI) were used to evaluate the relationship obtained by multiple logistic regression. Of 1880 subjects who underwent US and a pathological diagnosis of CCA, the overall rate of CCA was 35.74%. CCA rate among those with liver mass (LM) was 54.85% and with dilated bile duct (DBD) was 62.01%. The relationship between DBD and CCA was highly significant (AOR = 3.46; 95% CI 2.74–4.36) followed by LM (AOR = 2.28; 95% CI 1.81–2.86) *P* value < 0.001. Our study reveals that US findings history have a strong association with CCA, especially in people diagnosed with DBD and LM. Therefore, these abnormalities can be indicators for suspected CCA diagnosis through US.

## Introduction

Hepatobiliary disorders are key indicators in the development of hepatocellular carcinomas (HCC) including cholangiocarcinoma (CCA), a fatal cancer of the bile duct^1–3^. Previous studies have shown various hepatobiliary abnormalities that were associated with CCA, such as a case–control study in China which reported cirrhosis and cholecystolithiasis are risk factors for intrahepatic CCA (ICC), and extrahepatic CCA (ECC), respectively^4,5^. A systematic review and meta-analysis showed that cysts and stones in the bile ducts were risk factors for ICC and ECC^6^. Moreover, a study in Mexico showed that primary sclerosing cholangitis (PSC) was a positive risk factor for developing ICC^7^, and a study in Finland also indicated that PSC was associated with both ICC and ECC^8^.

In Thailand, spatial analysis of hepatobiliary abnormalities in a high-risk population for CCA showed that the distribution of liver mass (LM), periductal fibrosis (PDF), and bile duct dilatation (BDD) were high in the lower and upper parts of the Northeast region, where CCA is also highly prevalent^9^. In addition, a study in Thailand conducted histopathological diagnosis and found PDF in CCA patients, this indicated a link between PDF, a serious bile duct disorder and CCA^10^. Another study in Thailand reported that BDD has also been found to be related with CCA^11^.

The incidence of CCA is increasing worldwide, and collectively CCA cancers comprise the second most common form of primary liver cancer^12,13^. However, the incidence of CCA cases is highly geographically concentrated, particularly within countries. For example, a previous study in Thailand, which has one of the highest national incidence levels globally, collected 39,421 CCA patients data between 2008 and 2013 and reported the annual incidence was highest in the Northeast region (62.8%)^14^. The rate of CCA is increasing every year, especially in the northeast of Thailand and is still a public health problem that needs to be addressed^14,15^.

Ultrasound (US) diagnosis is increasingly being used to detect hepatobiliary abnormalities associated with CCA risk, as well as early stage CCA, in order for treatment to be provided in a timely manner^16^. A study from a CCA screening program in Northeast Thailand concluded that US screening is an effective tool for detecting early stage CCA^17^. Studies have also supported US as an effective method for detecting early risk factors for CCA, particularly BDD^18,19^. Moreover, studies in Thailand indicate that US should be offered as a first screening tool for CCA^20^, and has an early CCA detection and surveillance role^10^.

Studies of CCA risk in Northeast Thailand to date have concentrated on other risk factors including: older age, infection with *Opisthorchis viverrini*, frequent praziquantel treatments, history of heavy alcohol consumption, history of smoking cigarettes, having diabetes mellitus, and environmental factors^21–24^. However, to date, few studies have examined connections between CCA diagnosis and a history of abnormalities of liver and bile ducts identified using US. Therefore, our study aimed to evaluate the rate of CCA and its association with the history of US findings in participants who participated in a large scale CCA screening program amongst a high risk population in Northeast Thailand.

## Materials and methods

### Data sources

Data from the Cholangiocarcinoma Screening and Care Program (CASCAP), Northeast Thailand were used for this study. CASCAP is the first project for large scale CCA screening in Thailand. The program has the aim of recruiting all residents aged 40 years and over in Northeast Thailand, a high risk area, for regular US screening for CCA and associated risk factors. The data resource profiles have been previously published^16,21^. In brief, the primary place for recruitment for the CASCAP study are 9 tertiary care hospitals in the Northeast region of Thailand. Subjects are recruited from these hospitals either from amongst patients who had presented with some symptoms that could be CCA related or from individuals present at hospital for other reasons and who were invited to join. All participants who underwent US through the CASCAP program, or those who underwent US elsewhere and were then enrolled in the program, between 2013 and 2020 were considered for inclusion in this study.

### Study population

**Figure 1:**
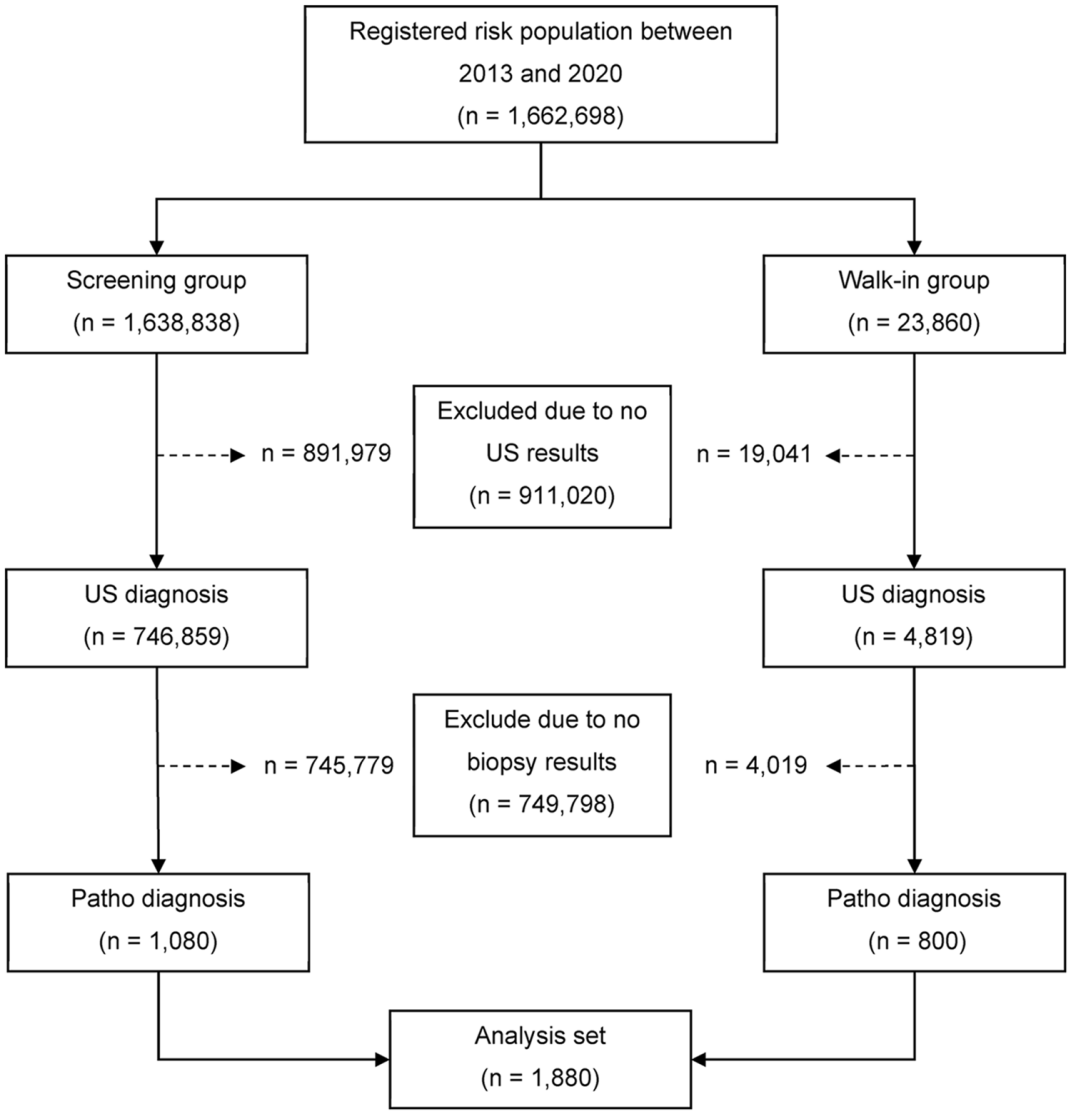
Sample selection process. US—Ultrasonography.

Our study includes people who participated in CASCAP from two cohort groups (screening and walk-in). The screening group were people who were attending health services for reasons unrelated to CCA, but who were invited to undergo US screening due to being in the target population. The walk-in group were people who presented at hospital with symptoms indicating CCA, and who were then assessed using US. The participants who have liver mass (LM) and/or dilated bile duct (DBD) from US findings were classified as having suspected CCA, and were referred to computed tomography (CT) or magnetic resonance imaging (MRI) to confirm the diagnosis. Where US, and CT or MRI diagnosis indicated CCA risk, both screening and walk-in groups, underwent pathological diagnosis. The subjects included in our study were those enrolled in the CASCAP database from 2013 to 2020, for whom US diagnosis indicated CCA risk, and who underwent pathological assessment for CCA, with a total of 1880 subjects (Fig. 1).

### Outcome and independent variables

The study outcome was CCA status from pathological diagnosis based on biopsy, which was initially categorized into four groups: not CCA, early stage, late stage, and stage unknown. Early stage of CCA was defined from stage 0, I, II and late state was defined from stage III and IV according to the 7th edition AJCC staging ^25,26^. Then, CCA data was categorized into two groups (no/yes) which was the primary outcome for this study. The final assignment of CCA stage in our study was based on the findings after completion of surgery or treatment where the clinical and/or pathological characteristics can be correctly determined. The data on histological findings were based on the standard protocol of the tertiary hospital at Khon Kaen University. The independent variables included cohort types (screening/walk-in), gender (female/male), and age at enrollment in years. These were collected using an enrolment questionnaire. The history of US findings: periductal fibrosis (none-PDF/PDF1/PDF2/PDF3), fatty liver disease (FLD) (no/yes), liver parenchymal change (LPC) (no/yes), liver mass (LM) (no/yes), and dilated bile duct (DBD) (no/yes), were collected from case record forms.

### Statistical analysis

The baseline characteristics and history of US findings were summarized using frequency counts and percentages for categorical data (i.e. cohort types, gender, age groups, PDF, FLD, LPC, LM, and DBD). The mean with standard deviation (SD), median, minimum and maximum range were summarized for continuous data such as age at enrollment in years. The rate of CCA was calculated by using the number of all CCA cases as the numerator and the total number of all study subjects who underwent US and histological examination as the denominator. This was calculated overall and separately by each factor category. Associations between explanatory factors and CCA were measured by crude odds ratios (ORs) using simple logistic regression. Stratified analysis was used to investigate the effect of each factor on the association between other factors using a Mantel–Haenszel test. The adjusted OR (AOR) and 95% confidence intervals (CIs) were obtained by multivariable analysis which was used to identify the association of all factors with CCA adjusted for the factors indicated above using multiple logistic regression. A *P* value of less than 0.05 was considered to be significant. All analyses were performed using STATA version 15 (StataCorp, College Station, TX).

### Ethical considerations

The research protocol was approved by Khon Kaen University Ethics Committee for Human Research, reference number HE621288. The data were provided from the Cholangiocarcinoma Screening and Care Program (CASCAP). The CASCAP data collection was conducted according to the principles of Good Clinical Practice, the Declaration of Helsinki, and national laws and regulations about clinical studies. It was approved by the Khon Kaen University Ethics Committee for Human Research under the reference number HE551404. All patients gave written informed consent for the study.

## Results

### Sample characteristics

A total of 1,880 subjects who underwent US and pathological diagnosis were enrolled in our study. More than half (57.4%) were from the screening group, males (59.9%), and aged at 60 years and over at enrolment (55%), with a mean age of 60.2 years (SD = 9.8). The history of US findings found around a quarter were diagnosed with LM (26.9%), and DBD (24.4%) (Table 1).

**Table 1:** Base line characteristics and ultrasound findings of participants in the CASCAP study 2013–2020.

Characteristics	Frequency	Percentage
**Cohort types**		
Screening	1080	57.4
Walk-in	800	42.6
**Gender**		
Female	753	40.1
Male	1127	59.9
**Age groups (years)**		
Less than 50	267	14.2
50–59	579	30.8
60 years and over	1032	55.0
Mean (standard deviation)	60.2 (9.8)	
Median (minimum:maximum)	61 (40:87)	
**Fatty liver disease**		
No	1683	89.5
Yes	197	10.5
**Liver parenchymal change**		
No	1720	91.5
Yes	160	8.5
**Periductal fibrosis (PDF)**		
No	1662	88.4
PDF1	122	6.5
PDF2	79	4.2
PDF3	17	0.9
**Liver mass**		
No	1375	73.1
Yes	505	26.9
**Dilated bile duct**		
No	1422	75.6
Yes	458	24.4

### Distribution of cholangiocarcinoma

**Figure 2:**
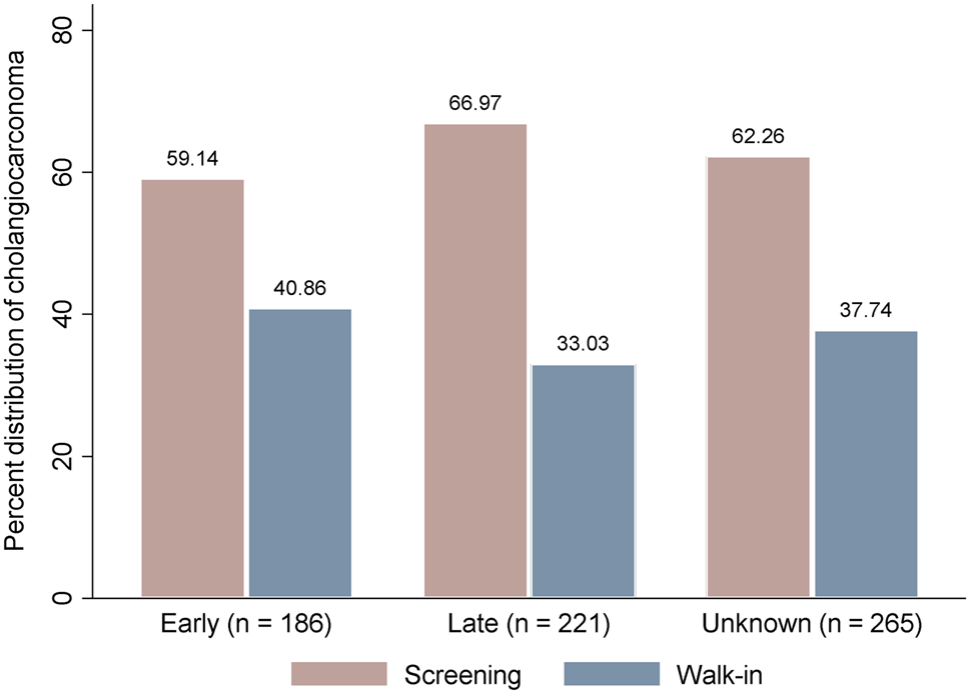
Percent distribution of cholangiocarcinoma according to cohort types. n—Number of cholangiocarcinoma patients.

Of 1,880 subjects, 672 were CCA positive (35.74%). From those CCA positive, the overall percent distribution of CCA categorized by staging, early, late, and unknown were 27.68%, 32.89%, and 39.43%, respectively (Table 2). Separated by cohort types, 423 CCA cases were from screening, and 249 cases were from the walk-in group. Around a quarter (26%) of the screening group, and around one-third of the walk-in group (30.52%) were in early stage of CCA. Figure 2 shows the distribution of CCA cases by stage according to screening and walk-in groups. There were 59.14% and 66.97% found in the screening group from 186 to 221 CCA cases at early and late stage, respectively.

**Table 2:** Percent distribution of cholangiocarcinoma according to staging and cohort group.

Stage of cholangiocarcinoma	All CCA cases *n* = 672		CCA in screening group *n* = 423		CCA in walk-in group* n* = 249	
	Number	(%)	Number	(%)	Number	(%)
Early	186	(27.68)	110	(26.00)	76	(30.52)
Late	221	(32.89)	148	(34.99)	73	(29.32)
Unknown	265	(39.43)	165	(39.01)	100	(40.16)

CCA cholangiocarcinoma, *n* number of sample, Unknown—Can not identify stage of CCA.

### Associations between history of ultrasonography findings and cholangiocarcinoma

**Figure 3:**
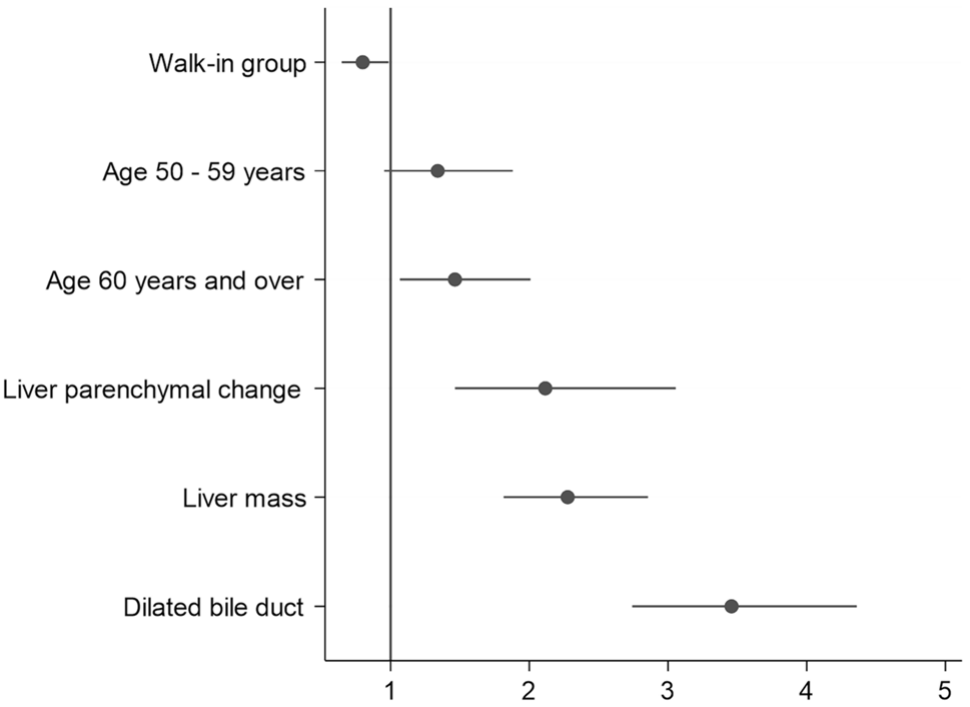
Odds ratios of the association between history of ultrasound findings and cholangiocarcinoma adjusted for cohort types and age of subjects at enrollment.

The overall rate of CCA was 35.74% from a total of 1880 subjects who underwent biopsy with a pathological diagnosis. Crude analysis using simple logistic regression found factors which had significant (*P* value < 0.05) associations with CCA were cohort types, age groups, LPC, LM, and DBD (Table 3). All of these variables were then included in the multivariable model measuring relationships between each factor and CCA using multiple logistic regression, also shown in Table 3. The results found that subjects from the walk-in group had less chance of having CCA compared to the screening group (AOR = 0.80; 95% CI 0.65–0.98; *P* value = 0.036), subjects aged from 60 years and over have 46% higher chance of having CCA compared to those aged < 50 years (AOR = 1.46; 95% CI 1.07–2.01; *P* value = 0.049). Compared to the normal group, subjects with hepatobiliary abnormalities diagnosed by US were more likely to have CCA, AOR of LPC was 2.12 (95% CI 1.46–3.06), LM was 2.28 (95% CI 1.81–2.86), and DBD was 3.46 (95% CI 2.74–4.36) with the *P* value < 0.001 for all factors (Table 3 and Fig. 3).

**Table 3:** Crude and adjusted odds ratio of association between ultrasound findings and cholangiocarcinoma using multiple logistic regression.

Factors	Number	CCA (%)	COR*	AOR	95% CI	*P* value
Over all	1880	35.74	N/A	N/A	N/A	N/A
**Cohort types**						0.036
Screening group	1080	39.17	1	1		
Walk-in group	800	31.13	0.71	0.80	0.65–0.98	
**Age groups (years)**						0.049
Less than 50	267	28.46	1	1		
50–59	579	34.72	1.34	1.34	0.95–1.88	
60 years and over	1032	38.18	1.55	1.46	1.07–2.01	
**Liver parenchymal change**						< 0.001
No	1720	32.97	1	1		
Yes	160	65.63	3.88	2.12	1.46–3.06	
**Liver mass**						< 0.001
No	1375	28.73	1	1		
Yes	505	54.85	3.01	2.28	1.81–2.86	
**Dilated bile duct**						< 0.001
No	1422	27.29	1	1		
Yes	458	62.01	4.35	3.46	2.74–4.36	

N/A not applicable, CCA cholangiocarcinoma, COR crude odds ratio, AOR Adjusted odds ratio, 95% CI—95% confidence interval of adjusted odds ratio; *P* value—Probability values from likelihood-ratio chi-square tests; *All crude odds ratio represent statistically significant association, *P* value < 0.05.

**Figure 4:**
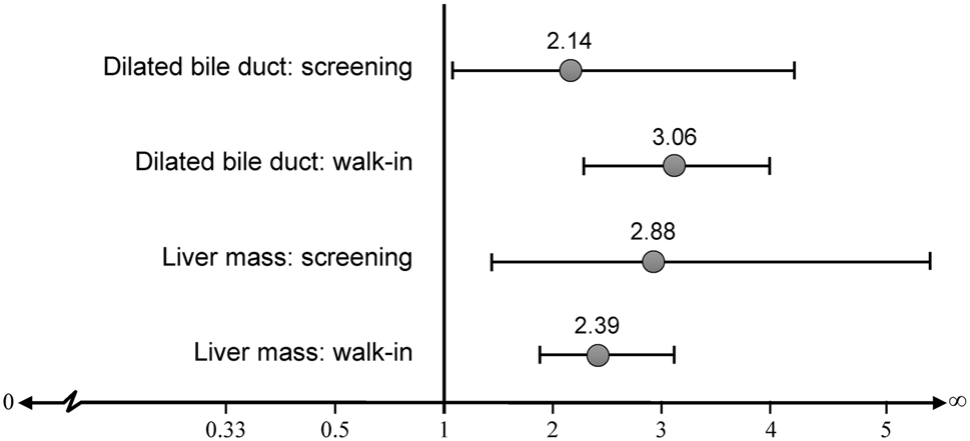
Odds ratios of interaction effects between cohort types and history of ultrasonography findings with cholangiocarcinoma association adjusted for age at enrollment, liver parenchymal change, cohort types, dilated bile duct, and liver mass.

Interaction effect results between history of US findings and CCA stratified by cohort types controlled for all factors such as age at enrollment, cohort types, LPC, DBD, LM, interaction between cohort types and DBD, and interaction between cohort types and LM were then calculated. The association between abnormal DBD and CCA was stronger in the walk-in group than in the screening group (AOR = 3.06; 95% CI 2.29–4.09; *P* value < 0.001, and AOR = 2.14; 95% CI 1.07–4.28; *P* value = 0.032, respectively). However, the association between LM and CCA was higher in the screening group (AOR = 2.88; 95% CI 1.46–5.67; *P* value = 0.002, and AOR = 2.39; 95% CI 1.81–3.15; *P* value < 0.001, respectively) (Table 4 and Fig. 4).

**Table 4:** Interaction between cohort types and history of ultrasonography findings with cholangiocarcinoma association using multiple logistic regression.

Factors	Number	CCA (%)	COR	AOR	95% CI	*P* value
Bile duct dilatation finding according to cohort types						
**Screening group**						0.032
None-dilated bile duct	793	31.27	1	1		
Dilated bile duct	287	60.98	3.43	2.14	1.07–4.28	
**Walk-in group**						< 0.001
None-dilated bile duct	629	22.26	1	1		
Dilated bile duct	171	63.74	6.14	3.06	2.29–4.09	
Liver mass finding according to cohort types						
**Screening group**						0.002
None-liver mass	735	31.43	1	1		
Liver mass	345	55.65	2.74	2.88	1.46–5.67	
**Walk-in group**						< 0.001
None-liver mass	640	25.62	1	1		
Liver mass	160	53.13	3.29	2.39	1.81–3.15	

*CCA* cholangiocarcinoma, *COR* crude odds ratio, *AOR* adjusted odds ratio, 95% CI—95% confidence interval of adjusted odds ratio; *P* value, probability values from likelihood-ratio chi-square tests.

## Discussion

Our study evaluated the rate of CCA and its association with history of liver and bile duct disorders diagnosed with US^1–3^, in a large scale CCA screening program (CASCAP) in a CCA high-risk population, Northeast of Thailand^16^. Our study findings indicate a high overall proportion of positive CCA cases which was consistent with previous studies indicating that Thailand has the highest incidence of CCA in the world^15^, especially in the Northeast region^14^. The most important finding of this study is the strong relationship between adverse US results and subsequent CCA diagnosis, emphasising the importance of US screening in discovering early symptoms that could lead to more serious diseases, including CCA.

One of the most important findings of the paper is that subjects from the screening group have higher odds of CCA than those from the walk-in group. That is, active screening from the general population was 1.25 times more likely to identify cases of CCA. This could be because the general population of Northeast Thailand is already at high risk of CCA and large scale screening of this at-risk population was able to identify more cases than passive screening of the walk in group, and these cases were more likely to be early stage increasing chances of treatment and survival. As well, many of the CCA symptoms are fairly non-specific. So, the walk-in group with potential CCA symptoms such as malaise and jaundice, may in fact be suffering from other conditions. These findings are in line with the previous studies conducted on CCA screening in Thailand that found a high rate in this group^16,20^. This has important implications for the design of programs to reduce the mortality rate from CCA.

The rate of CCA increased with age in our results, subjects aged higher than 50 years were more likely to have CCA compare to those aged less than that. This result similar to a study on risk factors for CCA in 2022 from USA which found that CCA patients had a mean age of 67 years (SD = 12.8 years)^27^. This is because the development of CCA takes time. Almost all people in the region have a habit of eating raw fish leading to risk of infection with the liver fluke *Opisthorchis viverrini* which is one of the main causes of CCA in Thailand. Therefore, after infection with *O. viverrini*, the parasites will live in the liver and bile duct for about 20–30 years until CCA develops^28^.

Our results also found that there was a high association between subjects diagnosed with LPC, LM, and DBD and CCA. These relationships were consistent with previous studies indicated that abnormalities of liver and bile duct were associated with CCA^10,18,19^. Separately for the screening and walk-in groups, our results found a high association between history of US findings of DBD and LM, and CCA, the study area has a high prevalence of hepatobiliary abnormalities^9^. Our study also found that in the walk in group DBD was highly associated with CCA, whereas in the screening group the relationship was stronger for LM. However, these effects were in the same direction with the adjusted OR = 3.06, 95% CI 2.29–4.09, and adjusted OR = 2.88, 95% CI 1.46–5.67 for both DBD and LM, respectively. Both DBD and LM are important indicators of CCA and can be detected through US screening according to previously reported studies^18,19,29^. Other studies have been conducted in the northeastern region of Thailand. In 2019 Chamadol and colleges reported a relationship between PDF and BDD, which is associated with CCA, and PDF can also be an indicator for suspected CCA diagnosis through US^30^. Also in 2020, Khuntikeo and colleges reported US screening is an effective tool for detecting early stage of CCA in high incidence areas^17^. In addition, there was US screening for CCA program conducted in the north of Thailand in 2016 by Sungkasubun and colleges to detect early stage of CCA which recommended that US should be offered as a first screening tool for CCA in an endemic area^20^.

The limitation of our study is that the temporal sequence of disease development cannot be clearly identified, between liver and bile duct abnormalities and CCA. As we cannot define clearly that these abnormalities preceded CCA development we cannot identify the exact cause of CCA. Therefore, we can only report such factors as being associated with CCA. In addition, the high rates of hepatobiliary abnormalities identified in our study subjects is likely due to the high risk population and cannot represent disease burdens in other areas of Thailand, or internationally.

However, our study has a sufficient sample size, allowing us to achieve a precise estimate of the OR leading to conclusive findings. Further studies should be conducted in population that are diverse in both behavioral and geographic areas. Prospective studies are needed to monitor healthy individuals until intra-abdominal abnormalities are identified and eventually progress to CCA.

## Conclusions

Our study findings demonstrated that hepatobiliary abnormalities detected by US were associated with CCA, especially in subjects diagnosed with DBD, and LM which were strongly related to CCA. These liver and bile ducts abnormalities can be very important indicators for CCA. Therefore, we recommend that screening for CCA in populations living in high-risk areas, as well as the general population, should focus on finding early symptoms to detect suspected CCA that could develop into CCA such as liver and bile ducts abnormalities through US diagnosis.

## Data Availability

The datasets generated during and/or analyzed during the current study are available from the corresponding author on reasonable request.
